# Metabolic reprogramming in tumor-associated cells of hematologic malignancies: mechanisms, crosstalk networks, and therapeutic implications in the tumor microenvironment

**DOI:** 10.3389/fimmu.2026.1773233

**Published:** 2026-03-24

**Authors:** Zikun Hong, Yuming Wu, Wenqi Liu, Dehu Li, Jian Xu

**Affiliations:** 1 Institute of Hematology, Union Hospital, Tongji Medical College, Huazhong University of Science and Technology, Wuhan, China; 2 Department of Hematology, The Second Affiliated Hospital, College of Medicine, Zhejiang University, Hangzhou, Zhejiang, China

**Keywords:** amino acid metabolism, glucose metabolism, hematologic malignancies, lipid metabolism, metabolic reprogramming, tumor microenvironment, tumor-associated cells

## Abstract

Hematologic malignancies (HMs), which originate from hematopoietic or lymphoid tissues, pose a significant therapeutic challenge due to issues such as drug resistance, relapse, and treatment-related toxicity. The tumor microenvironment (TME), especially within the bone marrow niche, is now widely recognized as a critical determinant of disease progression and treatment response. A central mechanism within this specialized niche is the extensive metabolic reprogramming of key stromal and immune cells, including tumor-associated macrophages (TAMs), myeloid-derived suppressor cells (MDSCs), cancer-associated fibroblasts (CAFs), and bone marrow adipocytes (BMAds). This review systematically elaborates on the alterations in glucose, lipid, and amino acid metabolism within these cellular compartments of the HM-TME. We detail how metabolites such as lactate, fatty acids, and itaconate function not merely as metabolic byproducts but as active signaling molecules that drive critical processes like immune cell polarization, stromal remodeling, and intricate metabolic crosstalk. This comprehensive reprogramming collectively fosters a profoundly immunosuppressive milieu, promotes tumor cell survival and proliferation, and confers resistance to conventional and novel therapies. Furthermore, we explore emerging therapeutic strategies designed to target these metabolic vulnerabilities. These include inhibitors of specific metabolic pathways, modulators of metabolite-driven signaling, and innovative approaches such as nanomedicine and metabolically enhanced immunotherapy. Finally, we outline the current challenges in the field—such as intra-tumoral metabolic heterogeneity and the pressing need for targeted delivery systems—and discuss future perspectives involving advanced technologies like single-cell metabolomics and rational combination strategies. In summary, this synthesis aims to provide a comprehensive and rational foundation for developing novel immunometabolic interventions against HMs, highlighting the therapeutic potential of disrupting the metabolic dialogue within the TME.

## 1 Introduction

Hematologic malignancies (HMs) represent a heterogeneous group of cancers originating from the hematopoietic or lymphoid tissues, characterized by the clonal expansion of aberrant cells that disrupt normal hematopoiesis and immune function (1). Classically categorized into leukemias, lymphomas, multiple myeloma(MM), and myelodysplastic syndromes (MDS), these malignancies constitute a significant global health burden, remaining a leading cause of cancer-related morbidity and mortality (2). Although significant advances have been made in chemotherapy, molecularly targeted agents, and immunotherapy, the clinical management of these diseases continues to face persistent challenges. Issues such as intrinsic and acquired therapeutic resistance, disease relapse, and treatment-related toxicity frequently undermine long-term outcomes (3). These hurdles underscore the critical need to elucidate the complex pathobiological mechanisms—including those within the tumor microenvironment(TME)—that drive disease progression and therapy failure (4). A deeper understanding of these mechanisms is indispensable for developing the next generation of more effective and durable therapeutic paradigms.

The TME is a dynamic and complex ecosystem comprising malignant cells, diverse stromal and immune cell populations, extracellular matrix components, and unique physicochemical conditions (5). It is now widely recognized as a pivotal determinant of cancer progression and treatment response across malignancies, including HMs (6). While the classic”seed and soil” hypothesis applies broadly in oncology, the specific “soil” for HMs is fundamentally distinct from the spatially constrained and steep physicochemical gradients of solid carcinomas. The hematologic malignancy TME (HM-TME) is primarily housed within the specialized, ecosystem-like niches of the bone marrow (BM), lymph nodes, and secondary lymphoid organs—sites intrinsically responsible for normal hematopoiesis and immune cell development (6). Central to this unique ecosystem is the BM niche, a physiologically hypoxic(1–3% O_2_) sanctuary that malignant cells adeptly exploit to preserve stemness and evade therapy-induced oxidative stress (7).This setting is built upon three interconnected pillars that distinguish HMs from solid malignancies, and collectively define HM pathogenesis and therapeutic resilience.

**Figure 1:**
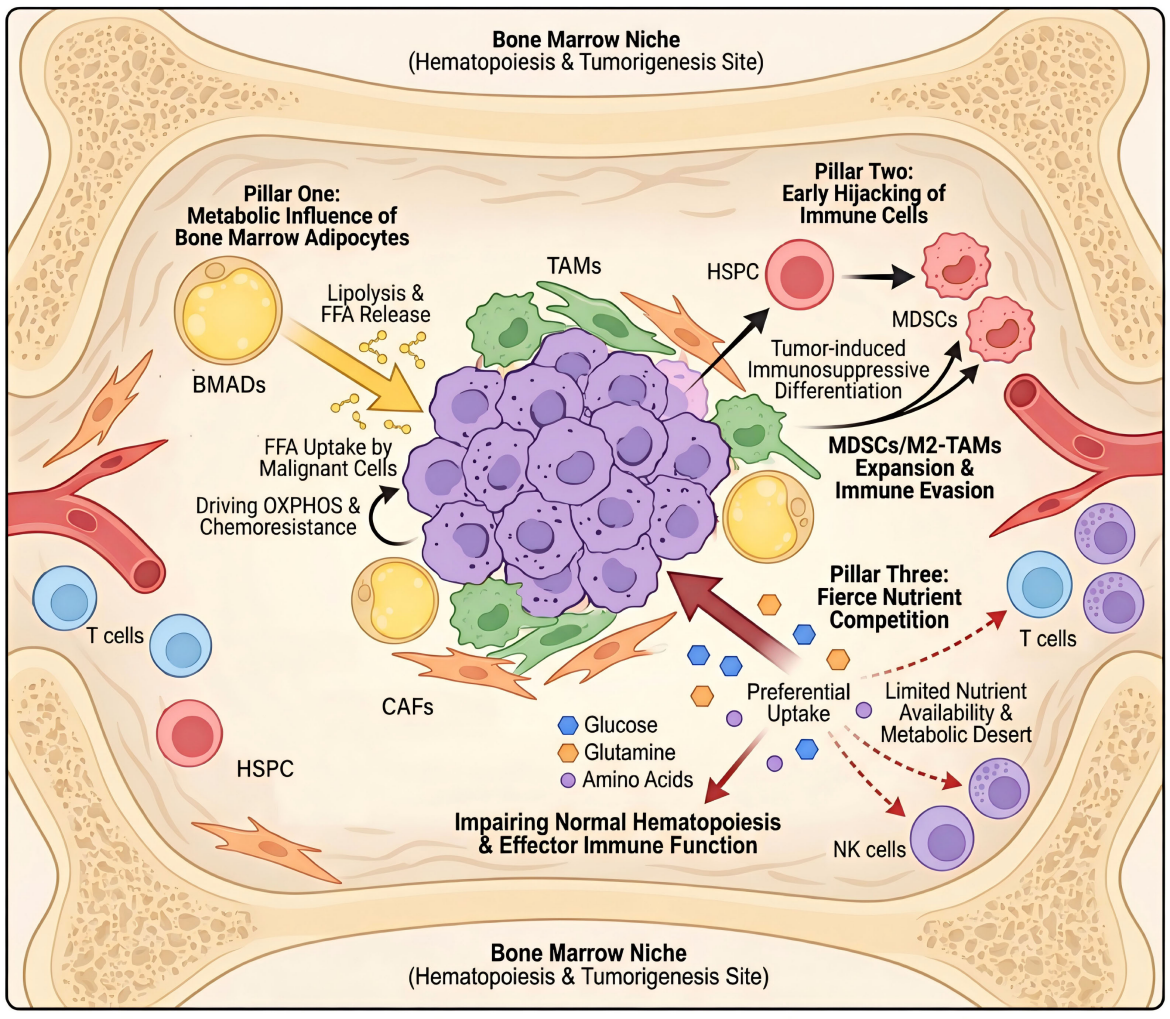
Distinct metabolic ecosystem of the hematologic malignancy tumor microenvironment (HM-TME). The unique metabolic ecosystem within the HM-TME, where metabolic reprogramming by bone marrow adipocytes (BMAds), pathological hijacking of immune cells, and fierce nutrient competition collectively establish an immunosuppressive milieu and drive tumor progression. Pillar one: Metabolic influence of BMAds. BMAd is a central metabolic regulator. BMAds undergo lipolysis to release free fatty acids (FFAs), which are preferentially taken up by malignant cells to fuel oxidative phosphorylation (OXPHOS) and enhance chemoresistance. Cancer-associated fibroblasts (CAFs) and tumor-associated macrophages (TAMs) contributes to this metabolic remodeling. Pillar two: Early hijacking of immune cells. Within the bone marrow niche, tumor cells subvert normal hematopoiesis. Hematopoietic stem and progenitor cells (HSPCs) are driven towards immunosuppressive differentiation, generating abundant myeloid-derived suppressor cells (MDSCs) and M2-like TAMs. This process is a key feature of HM-TME-mediated immune evasion. Pillar three: Fierce nutrient competition. Malignant cells exhibit avid uptake of key nutrients—glucose, glutamine, and other amino acids—creating a local “metabolic desert.” This nutrient deprivation impairs the function of normal HSPCs, T cells, and natural killer (NK) cells, further crippling anti-tumor immunity. Abbreviations and symbols: Beige structure, bone marrow niche; Large yellow circles, BMAds; Small yellow circles, free fatty acids; Purple irregular clusters, malignant cells; Green amorphous shapes, TAMs; Orange spindle shapes, CAFs; Red circles, HSPCs; Red amorphous shapes, MDSCs; Blue circles, T cells; Purple circles, NK cells; Blue hexagons, glucose; Orange hexagons, glutamine; Small purple circles, amino acids. All abbreviations are defined in the legend text.

First, the metabolic influence of bone marrow adipocytes (BMAds). BMAds function as reprogrammable metabolic hubs within the niche. Upon interaction with malignant cells, they transform into tumor-associated BMAds. This transformation accelerates lipolysis, releasing free fatty acids that fuel oxidative phosphorylation (OXPHOS) in tumor cells. Concurrently, these adipocytes secrete adipokines and cytokines, actively shaping a pro-tumorigenic and therapy-resistant niche (8–10). Second, the aberrant interaction with developing immune cells. As a primary lymphoid organ, the BM provides a unique setting where malignant cells can aberrantly co-develop with and “hijack” nascent immune cells, such as myeloid-derived suppressor cells (MDSCs), at their very source. This process corrupts normal immune development, leading to systemic immune evasion characterized by the enrichment of immunosuppressive populations like M2-like macrophages and regulatory T cells (Tregs) (11–13).Third, fierce metabolic competition within the confined marrow cavity. Malignant cells, endowed with high metabolic plasticity, aggressively outcompete normal hematopoietic and immune cells for scarce nutrients such as glucose and glutamine. This creates a local “metabolic desert” that cripples normal tissue function and impairs effector immunity. Crucially, these three pillars are not isolated but are integrated through profound metabolic-immune crosstalk. The functional executors of this crosstalk are diverse tumor-associated cells (TACs), including tumor-associated macrophages (TAMs), MDSCs, cancer-associated fibroblasts (CAFs), and others. Their phenotypes and functions are profoundly reshaped by the malignant niche, forming a cohesive, pro-tumor network (14–16) (**Figure 1**).

Metabolic reprogramming—a hallmark of cancer where cells rewire pathways for energy and biomass production—is not merely a cell-autonomous trait but is dynamically shaped by and, in turn, shapes the TME. The pro-tumorigenic functions of the HM-TME are intimately linked to its metabolic landscape. The hallmark features of this microenvironment — namely, a hypoxic background, a dependency on BMAds as key metabolic partners, a state of fierce nutrient competition, and the overarching goals of immunosuppression and therapy resistance — collectively subject TACs to profound metabolic reprogramming (15). For instance, this reprogramming is a key driver in polarizing macrophages toward an immunosuppressive M2-like phenotype, enhances MDSC suppression via fatty acid oxidation (FAO), and depletes nutrients to exhaust effector T and Natural Killer (NK) cells while stabilizing Tregs, thereby transforming the BM into an “immunological desert” (17, 18). Specific metabolic alterations, such as heightened glycolysis and glutaminolysis, are evident across HMs and support tumor cell proliferation, survival, and therapy resistance. Furthermore, amino acid metabolic reprogramming has been shown to intricately modulate tumor progression through interactions with key signaling pathways. Lipid metabolic reprogramming is particularly pivotal, influencing not only tumor cell proliferation but also immune cell function and the overall immunosuppressive landscape (19).

Despite growing recognition of the unique structural, immune, and metabolic features of the HM-TME, current understanding of its metabolic landscape remains fragmented. There is a notable lack of integrated analysis regarding the complex metabolic crosstalk among its diverse cellular constituents. Existing studies often focus on tumor cell-intrinsic metabolism or on isolated immune cell types, without synthesizing how metabolic reprogramming across key TACs—stromal (e.g., CAFs, BMAds) and immune (e.g., TAMs, MDSCs) cells—orchestrates a cohesive, pro-tumor network. Therefore, this review will focus on elucidating the metabolic reprogramming of key stromal and immune cells within the HM-TME. We will delve into their specific alterations in glucose, lipid, and amino acid metabolism, explore the underlying regulatory mechanisms, and critically examine how these shifts collectively promote tumor progression, immune suppression, and therapy resistance. By synthesizing existing literature, we aim to map the therapeutic vulnerabilities within these metabolic pathways and provide a rational foundation for developing novel, combined immunometabolic strategies against HMs.

## 2 Mechanisms of glucose, lipid, and amino acid metabolic reprogramming in the HM-TME

### 2.1 Glucose metabolism: a metabolic hub driving immunosuppression

Glucose metabolic reprogramming serves as a central organizational principle within the bone marrow microenvironment (BMM) of HMs, orchestrating both tumor progression and the immunosuppressive landscape.The concept of glucose metabolic reprogramming in tumor cells dates back to 1924, when German biologist Otto Warburg proposed that cancer cells preferentially generate ATP via glycolysis rather than OXPHOS, even under adequate oxygen—termed the Warburg effect. However, recent advances in understanding glucose metabolism reveal that cancer cells in certain HMs, such as specific mutant subtypes of acute myeloid leukemia (AML) and MM, remain highly dependent on mitochondrial respiration (20, 21). Notably, metabolic reprogramming is not confined to malignant cells. Numerous TACs within the TME, including stromal and immune cells, also rewire their glucose metabolism to execute pro-tumorigenic functions, adapting to the unique pressures of the BMM.

The BM is a highly vascularized yet physiologically hypoxic organ. During the progression of leukemia, MM, and lymphoma, this hypoxia is pathologically exacerbated. Within this niche, malignant cells engage in fierce metabolic competition with surrounding TACs, exhibiting exceptionally high glucose uptake rates. This competition plunges the local microenvironment into a state of glucose deprivation and leads to excessive lactate accumulation (22, 23). This extreme metabolic pressure forces TACs to undergo a metabolic shift, often from physiological OXPHOS to pathologically enhanced glycolysis, as an adaptive survival strategy within the hypoxic and nutrient-depleted BMM (24). This reprogramming has profound functional consequences. For example, it drives the polarization of TAMs and the formation of a glycolysis-dependent immunosuppressive phenotype of MDSCs. A key outcome is the significant lactate accumulation within the TME. Lactate is no longer viewed merely as a metabolic waste product, it acts as a potent signaling molecule and mediates lactylation modifications (e.g., histone lactylation). This modification serves as an epigenetic regulators of metabolism, directly linking metabolism to gene expression programs that support tumor immune evasion (25, 26). Beyond lactate, other specific metabolites produced by glucose metabolism of TACs, such as itaconate and α-ketoglutarate (α-KG), function as signaling molecules, providing feedback to regulate the metabolism and function of themselves or other cells. This metabolite interplay forms a key component of the regulatory network, constituting a complex web of metabolic crosstalk that defines the immunometabolic landscape of the TME (27).

This section will focus specifically on glucose metabolic reprogramming in TACs within the TME of HMs. We will dissect the upstream mechanisms that generate these specific metabolites, elucidate their downstream actions on various cell types, and analyze their impact on tumor progression. These reprogramming features are fundamental to TME cell adaptation and function. They are key drivers in shaping the immunosuppressive microenvironment, promoting tumor immune evasion and disease progression, making them thereby representing crucial targets for the discovery of therapeutic strategies.

#### 2.1.1 Lactate

Lactate accumulates substantially within the BMM of HMs, with concentrations typically ranging from 2 to 15 mM and reaching up to 30 mM in highly aggressive cases (28–30). Primarily, leukemic or myeloma cells produce and actively secrete massive amounts of lactate through aerobic glycolysis (31). Furthermore, the inherent hypoxia of the BM niche induces a metabolic shift from OXPHOS to glycolysis across various cell types (32). Additionally, bone marrow stromal cells (BMSCs), functionally reprogrammed by tumor cells, contribute to the extracellular lactate pool via the “Reverse Warburg Effect”, providing metabolic support to malignant cells while exacerbating local lactate accumulation (33). This metabolic niche, constructed by multi-source lactate, has emerged as a core organizational principle of BM niche-dependent metabolism in HMs, directly orchestrating subsequent immune evasion.

TAMs, immune cells infiltrating tumor tissues, primarily originate from the differentiation of circulating monocytes. Depending on microenvironmental signals, TAMs polarize into pro-inflammatory M1-type (anti-tumor) or anti-inflammatory M2-type (pro-tumor). M1 macrophages primarily execute anti-tumor functions by secreting IL-12 and TNF-α, and by highly expressing MHC Class II molecules to enhance antigen presentation and activate Th1 immune responses. Conversely, M2 macrophages are critical executors of immune suppression and tissue remodeling. At the molecular level, their phenotype is driven by core transcription factors such as STAT3 and Hypoxia-Inducible Factor-1α (HIF-1α). M2 TAMs suppress anti-tumor immunity by secreting inhibitory cytokines like IL-10 and TGF-β, and by highly expressing Arginase-1 (Arg-1), which depletes local arginine to suppress T cell function. Furthermore, they promote angiogenesis and matrix remodeling within the BM niche by secreting factors such as Vascular Endothelial Growth Factor (VEGF) and Matrix Metalloproteinase-9 (MMP-9) (34).

Lactate exerts profound regulatory effects on TAMs. In most cancers, lactate enters TAMs via monocarboxylate transporters (MCTs), activating HIF-1α and Nuclear Factor-κB (NF-κB) signaling pathways which induce expression of VEGF, arginase 1 (ARG1), and PD-L1, thereby driving TAM polarization toward the pro-tumorigenic M2 phenotype (35). Notably, lactate-mediated polarization follows consistent patterns in HMs. In diffuse large B-cell lymphoma (DLBCL), lactate promotes CXCL10 and PD-L1 expression in TAMs, driving Cytotoxic T Lymphocyte (CTL) exhaustion via the PD-1/PD-L1 axis, thereby impairing antigen presentation, weakening anti-tumor immune responses, and establishing an immunosuppressive microenvironment that accelerates DLBCL progression and chemoresistance (36, 37). Experimental studies in AML further demonstrate that lactate induces M2 polarization via G protein-coupled receptor 81 (GPR81), sustaining an immunosuppressive microenvironment (38). This GPR81-dependent signaling is a key mechanism in the polarization of leukemia-associated macrophages (LAMs), a unique subset of TAMs in the leukemic BMM, and directly supports leukemia cell growth.

In HMs, the enhancement of macrophages’ own glycolytic activity can also promote their polarization toward an M2-like, pro-tumorigenic phenotype. The expression of glycolytic genes in TAMs can be mediated by multiple pathways. For instance, in DLBCL, tumor-derived exosomal ENO2 activates the GSK3β/β-catenin/c-Myc axis, accelerating glycolysis in TAMs and reinforcing their M2 phenotype to promote tumor malignancy (39). Intriguingly, *in vitro* polarization assays reveal a more complex picture. The deficiency of BCL-3 upregulates glycolytic genes via NF-κB signaling and accelerates glycolysis, yet paradoxically enhances the expression of inducible nitric oxide synthase (iNOS), promoting M1 polarization and pro-inflammatory responses (40). This contradiction highlights the incomplete understanding of the precise mechanisms governing lactate-mediated TAM polarization and underscores the need for further investigation into the context-dependent roles of specific metabolic regulators. Overall, however, the prevailing evidence indicates that lactate robustly promotes M2 polarization in the TME.

Conversely, inhibiting lactate metabolism in macrophages can reverse their polarization. For example, in Hodgkin lymphoma (HL), the Phosphatidylinositol 3-Kinase δ/γ (PI3Kδ/γ) inhibitor RP6530 downregulates pyruvate kinase M2 (PKM2) in TAMs to reduce lactate production. This metabolic intervention reprograms TAMs from M2 to M1 phenotype, reshapes a pro-inflammatory TME, and suppresses angiogenesis and tumor proliferation (41).

MDSCs, expanded populations of immature myeloid cells in the TME, originate from common myeloid progenitors (CMPs) in the BM (42). Under normal physiological conditions, CMPs in the BM differentiate into mature granulocytes, monocytes/macrophages, and dendritic cells. However, within the TME, signals released by tumor cells, including granulocyte-macrophage colony-stimulating factor (GM-CSF), IL-6, and VEGF, persistently activate key signaling pathways such as JAK/STAT, NF-κB, and Notch. This disrupts normal myeloid development, impeding the maturation of myeloid precursors. Consequently, these cells proliferate extensively and differentiate into highly immunosuppressive MDSCs (43). Based on morphology, phenotype, and function, MDSCs are primarily classified into two subpopulations: granulocyte-like MDSCs (PMN-MDSCs) and monocyte-like MDSCs (M-MDSCs). Despite mechanistic differences, both subtypes foster immunosuppression by depleting arginine (via ARG1/iNOS), generating reactive oxygen species (ROS)/nitric oxide(NO) to impair T/NK cell cytotoxicity, and expanding Tregs, thereby serving as central hubs for immune evasion (44). Furthermore, M-MDSCs exhibit greater plasticity. They can not only further differentiate into TAMs within the TME but also indirectly suppress immune responses by inducing the expansion of Tregs through the secretion of cytokines such as IL-10 and TGF-β (45).

Lactate is closely associated with the expansion and function of MDSCs within HMs. In MM, lactate has been shown to induce the expansion of M-MDSCs and Tregs via non-GPR81 pathways, thereby reshaping the local immunosuppressive niche (46). In contrast, the GPR81-dependent signaling axis of lactate is more extensively characterized in solid tumors. For instance, in colorectal cancer, lactate activates GPR81 on tumor cells, leading to the upregulation of chemokines CCL2/CCL7 via the 14-3-3/Signal Transducer and Activator of Transcription 3 (STAT3) pathway.This chemokine gradient recruits CCR2^+^ PMN-MDSCs into the TME, which subsequently inhibit the function of CD8^+^ T cells (47). Additionally, lactate contributes to an acidic TME by lowering extracellular pH (48), a condition that indirectly enhances MDSC recruitment and activation while impairing the cytotoxicity of T cells and NK cells through mechanisms involving the production of ROS and NO (49).

CAFs, activated stromal cells derived from resident fibroblasts or mesenchymal stem cells, remodel the extracellular matrix via collagen and fibronectin secretion and directly stimulate tumor proliferation, invasion, and stemness through CXCL12, HGF, and FGF. They also recruit immunosuppressive cells, collectively driving therapy resistance (50). The “Reverse Warburg Effect” characterizes metabolic shifts in CAFs, wherein OXPHOS is replaced by aerobic glycolysis (51). Validated in solid tumors, this model posits that CAFs fuel tumor cells by supplying lactate and pyruvate, enhancing aggressiveness (52). Consistently, CAFs in HMs exhibit similar alterations. Studies in malignant lymphomas show CAF-secreted pyruvate is imported by lymphoma cells via MCTs, enriching tricarboxylic acid (TCA) cycle intermediates to support aerobic metabolism and survival (53). Furthermore, the reliance of AML cells on the TCA cycle for energy production, rather than the classic Warburg effect further supports the existence of this metabolic symbiosis in HMs, potentially mediated by mechanisms involving mitophagy or MCT4 overexpression (54–56). This metabolic coupling between tumor and stroma, confirmed in DLBCL, strongly correlates with enhanced invasion, immune evasion, and chemoresistance (57). Recent studies have expanded our understanding of lactate’s role in shaping the stromal compartment. In myeloproliferative neoplasms (MPN), lactate accumulation has been found to induce the differentiation of healthy BMSCs toward a CAF-like phenotype, promoting collagen deposition—a process critical for fibrotic progression. Notably, this pro-fibrotic transformation can be significantly reversed by inhibiting MCT1-mediated lactate transport, highlighting a direct link between lactate metabolism and stromal remodeling in HMs (58).

Beyond its roles as a metabolic fuel and signaling molecule, lactate also functions as a substrate for a novel post-translational modification known as lactylation. This process involves the covalent modification of histone lysine residues by lactate, directly reshaping the transcriptional landscape and epigenetic regulation within cells, thereby adding another layer to lactate’s multifaceted influence on the TME.

In HMs, lactylation research has predominantly focused on tumor cells. Studies reveal that lactylation directly drives oncogene transcription and regulates cell fate through epigenetic mechanisms, serving as a key driver of disease development and progression. For instance, in T-cell acute lymphoblastic leukemia (T-ALL), H3K18la extensively enriches the promoters and enhancers of oncogenes such as NOTCH1 and TAL1 in T-ALL cells. These modifications form super-lactylation regions with properties akin to super-enhancers which drive malignant transcriptional programs and correlate with poor prognosis (59). Similarly, in MM, knockdown of the lactylation-related gene PFN1 reduces the expression of CyclinD1, CDK4, and BCL2, leading to cell cycle arrest and apoptosis of MM cells (60). Beyond its direct effects on tumor cells, lactylation also participates in shaping the immunosuppressive TME. In AML, STAT5-driven lactate accumulation mediates H4K5la at the PD-L1 promoter via E3BP nuclear translocation in AML cells, thereby promoting CD8^+^ T cell exhaustion (61). These findings underscore the involvement of lactylation in multi-pathway regulation within hematologic TMEs, although its specific effects on other stromal cells components remain to be elucidated.

**Figure 2:**
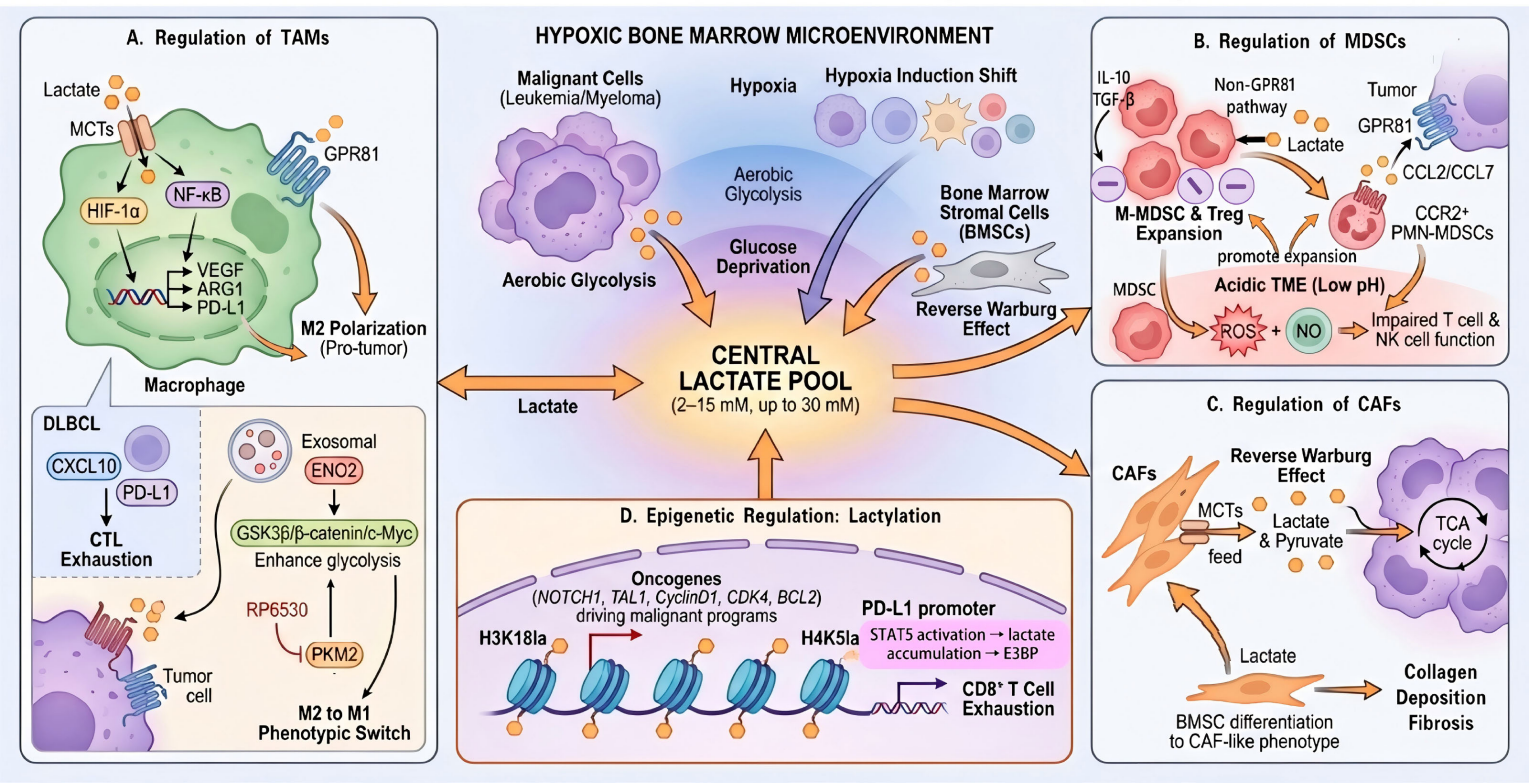
Multifaceted roles of lactate in the hematologic malignancy tumor microenvironment (HM-TME). This schematic illustrates the formation of a central lactate pool in the hypoxic hematologic malignancy tumor microenvironment and its profound impact on key tumor-associated cells (TACs), collectively fostering immunosuppression and disease progression. **(A)** Lactate reprograms tumor-associated macrophages (TAMs). Lactate enters TAMs via monocarboxylate transporters (MCTs), activating hypoxia-inducible factor 1-alpha (HIF-1α) and nuclear factor kappa-light-chain-enhancer of activated B cells (NF-κB) signaling to induce expression of vascular endothelial growth factor (VEGF), arginase-1 (ARG1), and programmed death-ligand 1 (PD-L1). Lactate also directly binds G protein-coupled receptor 81 (GPR81), collectively driving TAMs toward a pro-tumorigenic M2 phenotype. In diffuse large B-cell lymphoma (DLBCL), tumor-derived exosomal enolase 2 (ENO2) is taken up by macrophages, enhancing glycolysis via the glycogen synthase kinase 3 beta (GSK3β)/β-catenin/c-Myc axis and promoting pyruvate kinase M2 (PKM2) expression. The PKM2 inhibitor RP6530 can induce an M2-to-M1 phenotypic switch, augmenting cytotoxic T lymphocyte (CTL) activity and suppressing PD-L1 expression. **(B)** Lactate expands and activates immunosuppressive myeloid cells. Lactate promotes the expansion of monocytic myeloid-derived suppressor cells (M-MDSCs) and regulatory T cells (Tregs), potentially via GPR81 or alternative pathways. It also recruits CCR2^+^ polymorphonuclear MDSCs (PMN-MDSCs) via chemokine (C-C motif) ligand 2/7 (CCL2/CCL7). Within the acidic HM-TME, MDSCs generate abundant reactive oxygen species (ROS) and nitric oxide (NO), impairing the function of T cells and natural killer (NK) cells. **(C)** Lactate remodels the stromal compartment via cancer-associated fibroblasts (CAFs). Lactate enters CAFs via MCTs and is converted to pyruvate through the “Reverse Warburg Effect, “ fueling the tricarboxylic acid (TCA) cycle. Furthermore, lactate induces bone marrow stromal cells (BMSCs) to differentiate into a CAF-like phenotype, promoting collagen deposition and stromal remodeling. **(D)** Lactate acts as an epigenetic modifier via histone lactylation. Lactate mediates histone lactylation modifications, such as histone H3 lysine 18 lactylation (H3K18la) and histone H4 lysine 5 lactylation (H4K5la). For instance, H4K5la activates the signal transducer and activator of transcription 5 (STAT5) signaling pathway to enhance PD-L1 promoter activity, leading to CD8^+^T cell exhaustion. Abbreviations and symbols: High-concentration blue gradient, central lactate pool (2–15 mM, up to 30 mM); Purple irregular clusters, malignant cells (e.g., leukemia, myeloma, DLBCL); Light gray spindle cells, BMSCs; Orange spindle shapes, CAFs; Green amorphous shapes, TAMs; Red amorphous shapes, MDSCs; Purple circular cells, Tregs; Red small vesicles, exosomes. All abbreviations are defined within the legend text.

In summary, lactate in BMM of HMs is not merely a metabolic byproduct but a key organizer that reshapes TAC functions and constructs an immunosuppressive ecosystem. Lactate drives pro-tumorigenic M2 TAM polarization, promotes MDSC expansion and immunosuppression, and enables CAFs to fuel tumors via the Reverse Warburg Effect. Concurrently, lactate-mediated lactylation dysregulates the immune microenvironment by epigenetically upregulating PD-L1 in tumor cells to induce T cell exhaustion (**Figure 2**).

#### 2.1.2 Itaconate

Itaconate, an emerging focus in metabolic reprogramming research within the BMM of HMs, is a metabolite generated by the decarboxylation of the TCA cycle intermediate cis-aconitate, catalyzed by immune-responsive gene 1 (IRG1, encoding aconitate decarboxylase 1, ACOD1). Under physiological conditions, itaconate participates in the anti-inflammatory regulation of macrophages. Its levels are significantly elevated through metabolic reprogramming in TAMs, reshaping the functions of TACs in HMs (62).

Within the TME of HMs, itaconate is recognized as a key signaling molecule that drives the polarization of TAMs toward the M2 phenotype. Research demonstrates that itaconate derived from TAMs activates the nuclear factor erythroid 2-related factor 2 (NRF2) signaling pathway, thereby inducing antioxidant gene expression. This mechanism sustains the survival and immunosuppressive functions of M2 macrophages within the niche’s complex oxidative stress environment (63). This metabolically driven polarization directly leads to the secretion of high levels of IL-10 and TGF-β by TAMs, establishing a potent anti-inflammatory and pro-tumorigenic barrier in the BM. Furthermore, studies in HMs, using a B-cell lymphoma mouse model, revealed that inhibiting the pentose phosphate pathway in TAMs reduces itaconate levels. This attenuation diminishes the suppression of succinate dehydrogenase (SDH), leading to increased mitochondrial oxidative activity. The subsequent elevation in ATP-citrate lyase activity ultimately promotes macrophage activation, pro-inflammatory cytokine secretion, and a shift toward an M1-like phenotype (64).

Recent advances highlight itaconate’s reprogramming effects on MDSCs. In an EL4 lymphoma mouse model, β2-adrenergic receptor (β2-AR) signaling induced itaconate production in MDSCs. This itaconate maintained MDSC immunosuppressive function by enhancing NRF2 expression and controlling oxidative stress, thereby compromising doxorubicin efficacy against lymphoma (65).

Beyond its direct effects, itaconate acts as a key regulator in reprogramming other TACs and mediating complex intercellular communication within the TME, as well as between the TME and cancer cells. For instance, in a melanoma mouse model, itaconate secreted by MDSCs was taken up by CD8^+^ T cells. This uptake inhibited SDH activity and blocked aspartate synthesis, thereby impairing T-cell metabolism and cytotoxic function (66). Similarly, in non-small cell lung cancer (NSCLC), itaconate derived from TAMs stabilized the NRF2 protein, alleviated oxidative stress in cancer cells, and consequently enhanced resistance to radiotherapy. This metabolite-mediated crosstalk significantly contributes to tumor progression and therapy resistance (67). Intriguingly, the role of itaconate appears context-dependent and can be antagonistic toward cancer cells, particularly in HMs. In an EL4 mouse lymphoma model, thimerosal-induced, tumor cell-intrinsic biosynthesis of itaconate enhanced tumor immunogenicity by promoting transcription factor EB (TFEB)-mediated nuclear translocation and upregulation of antigen presentation-related genes (68). Furthermore, in chronic lymphocytic leukemia (CLL), treatment with the cell-permeable derivative dimethyl itaconate (DI) selectively abrogated metabolic activation and reduced viability of primary leukemic cells, even in the presence of protective microenvironmental signals (69).

It is important to note that the sources of itaconate in these studies varied, including endogenous synthesis within tumor cells and the application of exogenous derivatives. Therefore, the results require cautious interpretation regarding the net role of itaconate in HMs, as its effects may differ based on its cellular origin and concentration.

Beyond lactate and itaconate, other glycolytic and TCA cycle intermediates play indispensable roles in the metabolic crosstalk within the TME of HMs. For example, in MM, BMSCs can secrete metabolites like pyruvate to provide oxidative fuel for tumor cells. This metabolic symbiosis not only supports rapid proliferation but can also alter the local metabolic milieu, indirectly inducing dysfunction in surrounding immune cells (46). Succinate, another critical metabolite known to regulate the TME and influences invasiveness and drug resistance in HMs. In solid tumors, succinate binds to the macrophage-specific receptor SUCNR1, driving pro-tumorigenic M2 polarization via the PI3K/HIF-1α pathway (70). It can also modify proteins via succinylation to reshape cellular metabolism (71). However, succinate’s impact on TACs within the context of HMs, whether pro- or anti-tumorigenic, remains to be elucidated.

Collectively, glucose metabolic reprogramming in the HM-TME dynamically reshapes the immune landscape and drives malignant progression through metabolite interactions. Lactate, functioning as a central metabolic hub, induces M2 TAM polarization and MDSC expansion via the GPR81/HIF-1α axis. It also supplies energy substrates to tumor cells through the “Reverse Warburg Effect” in CAFs. Beyond its metabolic role, lactate directly influences gene expression by serving as a substrate for histone lactylation, thereby forming a “metabolic-epigenetic” pro-tumor network. In contrast, the role of itaconate appears more complex and context-dependent. While its impact on macrophage polarization is debated, itaconate unambiguously maintains the immunosuppressive function of MDSCs by enhancing NRF2 expression and controlling oxidative stress.This action synergizes with the effects of lactate to consolidate a state of immune escape within the TME. Nevertheless, the specific roles, precise mechanisms, and potential therapeutic implications of itaconate’s roles and mechanisms in the context of HMs, as opposed to solid tumors, demand deeper and more focused exploration.

### 2.2 Lipid metabolism: lipid signals reshape the microenvironmental architecture

Similar to glucose metabolism, the TME of HMs exhibits a unique and highly dynamic lipid metabolism landscape. This metabolic uniqueness partially stems from the abundant lipids within the BMM. BMAds, derived from mesenchymal stem cells, constitute a dynamic cellular reservoir that undergoes active turnover and secretes lipids into the niche. Concurrently, the high cellular activity, frequent apoptosis, and dense infiltration of immune cell within the BM collectively foster an environment characterized by intense lipid supply, consumption, and signaling. While lipid metabolic reprogramming in tumor cells is well-established, the reprogramming within TACs of the TME has recently emerged as a critical area of research (72).

TACs within the TME of HMs often exhibit upregulated expression of enzymes involved in lipogenesis. This reprogramming is mediated through key signaling pathways, including Adenosine Monophosphate-activated Protein Kinase (AMPK) and mammalian target of rapamycin (mTOR), peroxisome proliferator-activated receptor (PPAR), sterol regulatory element-binding transcription factor (SREBF), and LXR signaling pathways (73, 74). These pathways orchestrate lipid metabolism by increasing the expression of receptors (e.g., CD36) for exogenous lipid uptake or upregulating enzymes (e.g., FASN, ACLY) for *de novo* lipogenesis. Crucially, the lipid metabolites generated through these routes are not merely energy substrates, they also function as potent signaling molecules, providing feedback on cellular status and actively shaping intercellular communication within the TME (75, 76). This lipid metabolic reprogramming is particularly prominent in immunosuppressive cells such as M2 macrophages and MDSCs. Metabolites like fatty acids and lysophosphatidic acid, which accumulate during this reprogramming, actively participate in signaling crosstalk. This establishes a lipid metabolism-associated “energy base station” and contributes decisively to an immunosuppressive microenvironment (77). The following sections will delve into the sources, mechanisms of action on key tumor-associated immune cells, and ultimate impact on tumor biological behavior of several specific lipid metabolites playing crucial roles in the TME of HMs.

#### 2.2.1 Fatty acids

Fatty acids, serving as essential cellular energy sources, biomembrane constituents, and signaling molecule precursors, play a pivotal role in maintaining cellular homeostasis. Within tumor metabolic reprogramming, the aberrant activation of fatty acid metabolism, characterized by enhanced *de novo* synthesis, increased exogenous uptake, and altered oxidation, is a common feature across various HMs (78–82). Beyond fulfilling the bioenergetic and biosynthetic demands of rapidly proliferating tumor cells, fatty acids significantly contribute to TME metabolic reprogramming, thereby shaping tumor progression and therapeutic response.

A key mechanism through which fatty acids influence the HM-TME is by promoting an immunosuppressive landscape. Increased fatty acid uptake and transport can drive the polarization of TAMs toward an immunosuppressive M2 phenotype. For instance, in MM, IL-10 modulates macrophage metabolism via the PPARγ-fatty acid-binding protein 5 (FABP5) axis, augmenting lipid transport and accumulation to sustain M2 macrophage function and enhance chemoresistance (79). Furthermore, IL-10 also drives M2 polarization through the IL-10/IL-10R/STAT3 axis in macrophages, synergistically promoting MM immunosuppression and disease progression (80). This link between lipid metabolism and immune evasion is observed in other HMs as well. CD5-positive non-MYC/BCL2 double-expressor DLBCL exhibits activated fatty acid metabolic pathwaysalongside elevated proportions of M2 macrophage (81). Similarly, correlative studies suggest that poor prognosis in AML is associated with heightened fatty acid metabolism and extensive infiltration of M2 macrophages (82). Beyond supporting M2 polarization, specific fatty acids can directly reshape TAM function via signaling receptors, with polyunsaturated fatty acids (PUFAs) notably promoting the pro-inflammatory M1 phenotype in certain contexts. In MM, palmitic acid, a saturated fatty acid, was shown to upregulate arachidonate 12-lipoxygenase (ALOX12) expression in macrophages, and functional assays revealed that this upregulation inhibited AMPK phosphorylation in TAMs, blocked pro-apoptotic signal amplification, and reduced tumor cell sensitivity to proteasome inhibitors (83). Among TME-associated cells in HMs, BMAds exhibit a profound link to lipid metabolic reprogramming. BMAds are specialized adipocytes prevalent in the BM cavity that originate from BMSCs. Their formation is driven by PPARγ-mediated adipogenic differentiation, which involves the altered expression of PPARγ-, lipoprotein-, and adipokine-related genes. Conversely, the osteogenic differentiation of BMSCs, dominated by the transcription factor Runx2, entails upregulation of genes like alkaline phosphatase (ALP), osteocalcin, bone sialoprotein, and osterix, alongside downregulation of BMSC markers, ultimately yielding osteocytes. These adipogenic and osteogenic differentiation pathways are mutually inhibitory, creating a dynamic balance within the BM niche that can be co-opted by malignancies to support tumor growth (84).

BMAds are key promoters of tumor progression in HMs, primarily through the provision of fatty acids and secretion of adipokines (85). In MM, BMAd-released fatty acids fuel tumor growth and invasion, while adipokine-mediated interactions between MM cells and adipocytes enhance cancer cell proliferation and chemoresistance (86). In leukemia, a particularly potent crosstalk is observed. Tumor cells can secrete factors such as growth differentiation factor 15 (GDF15), which induces intense lipolysis in neighboring BMAds. The released free fatty acids are then taken up by leukemic cells, where they directly antagonize chemotherapy-induced mitochondrial apoptotic signals by activating survival pathways like Src/PI3K/AKT, thereby conferring significant drug resistance (87). While a minority of studies suggest BMAds may, in specific contexts, delay tumor progression -such as by impeding B-ALL cell engraftment and suppressing T-ALL proliferation *in vitro* and *in vivo* -the overwhelming body of evidence indicates their role is significantly pro-tumorigenic (88–91).

The formation of pro-tumorigenic BMAds is itself a process hijacked by malignancies. BMSC adipogenic differentiation is induced by diverse tumor-derived cytokines and signals. For instance, sterol regulatory element-binding transcription factor 1 (SREBF1) is a key mediator of dexamethasone-induced adipogenesis, which in turn provides metabolic support to residual T-ALL cells to enhance survival, chemoresistance, and risk of relapse. SREBF1 inhibitors can reduce this adipogenesis and its protective effect on leukemia (92). Ligand-receptor interactions, such as those involving TGFβ1, reshape the TME by shifting BMSC differentiation from osteoblasts to adipocytes, which then secrete growth factors that support MM expansion (93). Similarly, GREM1 deficiency promotes B-ALL proliferation and dexamethasone resistance by inducing BMSC adipogenesis via the BMP/SMAD pathway (89). Tumor-derived exosomes also play a critical role in redirecting BMSC fate. In AML, exosomal proteins like HNRNPM and FBL regulate BMSC differentiation and lipid reprogramming, and inhibiting exosome release has been shown to reduce adipogenesis and AML colonization in mouse models (94).

Once formed, BMAds undergo further tumor-driven remodeling to optimize their supportive functions. Epigenetic regulation, such as that mediated by the histone methyltransferase EZH2 represents a key mechanism. In MM, tumor cells activate ERK1/2 and NF-κB signaling in BMAds via integrin α6, leading to EZH2 upregulation. This promotes PRC2/SP1 complex-mediated PPARγ promoter methylation, causing an imbalance in adipokine secretion that favors tumor growth (95). MM cells also enhance m6A methylation of LncRNA within adipocyte-derived exosomes via EZH2-mediated methylation of METTL7A. This modification facilitates LncRNA binding to proteins like hnRNPA2B1 and hnRNPU for exosomal packaging.Once delivered, exosomal LOC606724 can bind to eIF4E, boosting c-Myc translation and inhibiting MM apoptosis (96), underscoring the pivotal role of exosomes in metabolic and epigenetic crosstalk.

Concomitant with the promotion of adipogenesis, tumors actively suppress the osteogenic lineage to create a more favorable niche. Reduced osteogenic differentiation and defects in BMSCs foster leukemia-supportive niches. For example, AML cells inhibit osteogenic transcription factors like RUNX2, in BMSCs via Notch signaling, thereby suppressing osteogenesis and promoting a pro-leukemic microenvironment (97). Similarly, AML-derived exosomes can induce DKK1 expression in stromal cells, which inhibits Wnt/β-catenin signaling, blocks osteoblast differentiation, and enhances AML proliferation and infiltration (98). These coordinated effects highlight the critical, bidirectional crosstalk between tumor cells and the BM stroma, which is essential for tumor survival and progression.In summary, within the BMM of HMs, fatty acids drive pro-tumor M2-like TAM polarization via enhanced uptake, transport, and oxidation, with effects varying across subtypes due to specific metabolite profile. BMAds themselves arise from tumor-induced adipogenic differentiation of BMSCs and subsequently support tumor proliferation, therapy resistance, and disease progression through a multifaceted program involving fatty acid provision, adipokine secretion, and epigenetic remodeling.

#### 2.2.2 Lysophosphatidic acid

Lysophosphatidic acid (LPA), a bioactive lipid mediator with growth factor-like properties, plays a pivotal role in regulating tumor cell proliferation, migration, survival, and metabolic reprogramming. It exerts its effects primarily by binding a family of six specific G protein-coupled receptors LPAR1-LPAR6. This engagement activates key downstream signaling pathways, including Rho/ROCK and PI3K/AKT, which collectively support the development and progression of cancer cells and TACs within HMs (99). Elevated levels of LPA in the TME primarily stem from enhanced synthesis and release by cancer cells themselves. However, contributions from other stromal components, such as BMAds and TAMs, are also significant (100). Through these sources, LPA signaling drives TME metabolic adaptation and broadly modulates the function of various TACs to promote HM progression.

As a key signaling lipid, LPA is a potent regulator of immune cell polarization. It mediates the polarization of TAMs towards M2-like phenotype, thereby facilitating the establishment of an immunosuppressive microenvironment. This process is receptor-specific. For instance, in Epstein-Barr virus (EBV)-associated B-cell lymphoma, secretory phospholipase A2 hydrolyzes tumor-derived extracellular vesicle (EV) phospholipids into LPA and PUFAs. LPA then promotes M2 polarization via LPAR1, accelerating lymphoma progression (101). Similarly, in ovarian cancer, LPA induces monocyte-to-M2 differentiation through LPAR1/3- mediated activation of the PI3K/AKT/mTOR-PPARγ signaling pathways. Concurrently, TAMs themselves can synthesize and secrete LPA, creating a feedforward loop that further enhances tumor invasion/metastasis (102). Overall, LPA is a potent driver of anti-inflammatory polarization and immunosuppression, modulated by microenvironmental factors.

Beyond immune modulation, LPA critically regulates the metabolic and functional reprogramming of CAFs. In MM, tumor cells induce mesenchymal stromal cells (MSCs) to secrete autotaxin (ATX), the key enzyme responsible for LPA generation. This ATX-generated LPA can then drive the transformation of MSCs into CAFs and stimulate the release of pro-angiogenic factors via LPAR1-NF-κB signaling. Interestingly, activation of a different receptor, LPAR3, may conversely inhibit this process, highlighting the complexity of LPA signaling (103).

The role of LPA extends to other immune components. Tumor-associated neutrophils (TANs), recruited to the TME, can polarize into pro-tumorigenic N2 phenotypes. These cells release extracellular matrix-degrading enzymes like MMP9, angiogenic factors, and T-cell suppressors such as ROS, PGE2, directly facilitating invasion/metastasis (104). Activated neutrophils can also form neutrophil extracellular traps (NETs)-DNA webs decorated with cytotoxic proteins (e.g., histones, myeloperoxidase). NETs promote metastasis by trapping circulating tumor cells and establishing immunosuppressive microenvironments via TLR9/NF-κB activation (105). Excessive NET formation is implicated in the progression of various HMs, including chronic myeloid leukemia, lymphoma, and MM, where it correlates with poor prognosis (106). Intriguingly, LPA appears to have a bidirectional modulatory effect on NETs, though this is context-dependent. In non-oncologic settings such as hyperlipidemic myocardial injury, LPA upregulation in monocytes can enhance NET-driven thromboinflammation (107).Conversely, in sepsis models, LPA has been shown to suppress NET formation (108).Whether LPA similarly regulates NETs dynamics within the HM TME to influence tumor invasion and immune evasion remains an important unanswered question.

#### 2.2.3 Sphingosine-1-phosphate

Sphingosine-1-phosphate (S1P) is a pleiotropic bioactive lipid mediator primarily generated by the phosphorylation of sphingosine, catalyzed by sphingosine kinases(SPHK). Upon its synthesis, S1P can be exported from cells and subsequently signals through G protein coupled receptors(S1PR1-S1PR5) in an autocrine or paracrine manner.This signaling axis regulates crucial processes in cancer progression, including cell proliferation, migration, survival, and metabolic reprogramming of tumor cells. Furthermore, S1P is a master regulator of immune cell trafficking and function, typically promoting an immunosuppressive environment by modulating lymphocyte egress from lymphoid organs and chemotaxis (109). Similar to other immunometabolites like itaconate, S1P significantly drives metabolic and functional reprogramming within the TME of HMs, with TAMs being key mediators. In DLBCL, tumor-derived S1P promotes monocyte and macrophage chemotaxis via S1PR1 while directly inhibiting the phagocytic capacity of M1 macrophages against antibody-opsonized tumor cells, thereby impairing the efficacy of anti-CD20 monoclonal antibody therapy.S1PR1 antagonists can reverse this immunosuppressive effect (110). Beyond chemotaxis, S1P can enhance arachidonic acid metabolism through pathways involving enzymes like ALOX15, thereby inducing M2-like macrophage polarization to further establish an immunosuppressive niche. This pro-tumorigenic rewiring of macrophages cooperates with other S1P-driven pathways, such as the S1P-YAP axis that promotes tumor cell invasion, to accelerate malignant progression, particularly in contexts like obesity-associated lymphoma (111).

As key signaling lysophospholipids, S1P and LPA exhibit functional and pathway synergy in promoting tumor growth and shaping the TME (112, 113). Critically, their metabolic pathways are intrinsically linked. Both originate from membrane phospholipid metabolism, their cellular levels are dynamically regulated by activation states, and they share common degradation enzymes like lipid phosphatases (114). Studies suggest a potential mutualistic relationship: in doxorubicin- resistant breast cancer models, LPA was shown to increase SPHK1 membrane translocation and activity through phospholipase D2 activation, thereby enhancing S1P synthesis and signaling to cooperatively promote cancer cell survival, metastasis, angiogenesis, and poor prognosis (115). However, the specific nature and significance of S1P-LPA crosstalk in remodeling hematologic TME remain to be fully elucidated. Therapeutic strategies concurrently targeting S1P and LPA signaling may prove more effective in disrupting their cooperative drive of immunosuppression and malignancy.

In summary, lipid metabolic reprogramming establishes dynamic signaling hubs within the hematologic TME, driving stromal ecotype restructuring and pro-invasive niche formation. Fatty acids promote M2 TAM polarization and MDSC immunosuppression via PPARγ signaling, while BMAds support tumor proliferation and therapy resistance through fatty acid and adipokine secretion. These fatty acids fuel oxidative metabolism in malignant cells and activate survival pathways such as Src/PI3K/AKT, while adipokines promote tumor cell proliferation and confer chemoresistance. LPA and S1P act as pivotal signaling lipids: LPA drives M2 TAM polarizationvia LPAR1/3-mediated NF-κB and PI3K pathways. S1P induces M2 and suppresses phagocytosis through mechanisms involving the S1PR1/ALOX15 axis, synergistically promoting lymphoma progression.Furthermore, The tight metabolic and functional linkage between S1P and LPA presents a combined promising therapeutic target for HMs.

### 2.3 Amino acid metabolism: nutrient deprivation and immune tolerance

Amino acid metabolism within the TME of HMs exhibits significant reprogramming, which is a critical driver of disease pathogenesis and therapy resistance. This metabolic rewiring is not merely a passive adaptation but an active process that underpins malignant core behaviors, including rapid and uncontrolled cancer cell proliferation, resistance to apoptosis, adaptation to the hypoxic and nutrient-depleted BM niche, and, most critically, the evasion of immune surveillance.

A hallmark of this reprogramming is the development of a heightened, and often essential, dependency on specific amino acids—a phenomenon termed “addiction”. The classic example is “glutamine addiction“, where tumor cells rely on exogenous glutamine not only as a nitrogen donor for nucleotide and amino acid biosynthesis but also as a crucial carbon source to replenish TCA cycle intermediates, thereby sustaining energy production and anabolic growth (116, 117). To meet this demand, the TME exhibits increased nitrogen demand, altered activity of key metabolic enzymes such as glutaminase (GLS) and arginase, and upregulation of amino acid transporters like ASCT2 (SLC1A5) for glutamine and LAT1 (SLC7A5) for branched-chain amino acids. Beyond their metabolic roles, amino acids act as signaling molecules, directly modifying oncogenic pathways such as Myc and mTOR, thereby entrenching the malignant state (118).

The impact of specific amino acids is diverse and context-dependent. Glutamine serves as a metabolic workhorse. After uptake and conversion to glutamate by GLS, it is transformed into α-KG to replenish the TCA cycle, providing biosynthetic precursors and energy. Arginine has a complex, dual role. While essential for cancer cell growth (e.g., for polyamine synthesis), its depletion in the TME by cells like MDSCs via ARG1 is a major mechanism of T-cell suppression, highlighting its function in both promoting and restraining tumors. Tryptophan metabolism is a cornerstone of immune evasion. Tumor and stromal cells catabolize tryptophan into kynurenine via enzymes like indoleamine 2, 3-dioxygenase 1 (IDO1), depleting the nutrient for effector T cells while generating an immunosuppressive metabolite that activates the aryl hydrocarbon receptor (AhR) (119, 120).Given this complexity, investigating amino acid reprogramming—not just in tumor cells but crucially within the diverse TACs of the TME—is of paramount importance. Decoding this “metabolic-immune-tumor network” is essential for developing novel therapies that can disrupt tumor support, reverse immunosuppression, and overcome resistance. The following sections will examine the impact of key dysregulated amino acids on different cellular components within the HM-TME.

#### 2.3.1 Glutamine

Glutamine, serving as the primary carrier for both carbon and nitrogen in the TME, undergoes profound metabolic reprogramming that dictates the functional evolution of TACs. In the progression of leukemia, lymphoma, and MM, TACs orchestrate glutaminolysis and its branched pathways to generate a spectrum of immunomodulatory metabolites, including glutamate, itaconate, succinate, and γ-aminobutyric acid (GABA). These metabolites serve dual purposes: they maintain the metabolic homeostasis of the TACs themselves and actively shape an immunosuppressive landscape through secretion and competitive nutrient uptake (121, 122).

Glutamine exhibits complex, bidirectional regulatory effects on macrophage polarization. It can promote an immunosuppressive M2 phenotype through mechanisms involving the glutamine-UDP-N-acetylglucosamine pathway and the resulting metabolite α-KG. Conversely, glutamine metabolism can also generate GABA, which can be further metabolized to produce succinate, a metabolite known to promote pro-inflammatory M1 polarization in certain contexts, such as in murine macrophages. However, within the specific context of HMs, the pro-tumorigenic, M2-polarizing effects of glutamine metabolism typically predominate, contributing to the immunosuppressive TME (123). The overall role of glutamine in dictating macrophage polarization remains an area of active investigation, with emerging evidence suggesting that a characteristic rewiring of glutamine metabolism is associated with M2 polarization. The dynamic equilibrium between glutamine anabolism, catalyzed by glutamine synthetase (GS), and catabolism, driven by GLS, appears to be a key determinant of TAM fate and function. Pharmacological inhibition of GS has been shown to shift M2 macrophages towards an M1-like phenotype, with significant immunological and functional consequences: enhanced ability of macrophages to recruit T cells, suppressed T cell inhibitory potential, inhibited endothelial cell branching (angiogenesis), and impaired cancer cell migration, collectively pointing to a therapeutic vulnerability that could be exploited to re-educate the TME (117, 124). However, the role of glutamine in macrophage polarization remains incompletely defined, with controversies arising from *in vitro* co-culture models, blockade of glutamine synthesis, and inhibition of glutamine uptake. These inconsistencies likely relate to the regulation of underlying metabolic flux balances or tumor type specificity, and the polarizing effect of glutamine on TAMs in HMs requires further validation.

In contrast, the relationship between glutamine and MDSCs, which share a myeloid origin and exhibit strong homology with TAMs, is more thoroughly investigated in lymphoma. Specifically, it reveals that the survival and functional persistence of MDSCs in HMs are highly dependent on their mitochondrial glutamine metabolism. In lymphoma microenvironments, β2-AR signaling axis activates the STAT3 pathway, enhancing mitochondrial glutamine metabolism in MDSCs. The resulting itaconate enhances the ROS scavenging capacity via NRF2-mediated oxidative stress responses, ensuring the long-term survival and efficient immunosuppressive activity of MDSCs within metabolically hostile BM niches. Importantly, it has also been shown to promote chemotherapy resistance in EL4 lymphoma and AML (65). Additionally, elevated granulocyte colony-stimulating factor (G-CSF) induce MDSC expression of γ-glutamyltransferase 1, hydrolyzing extracellular glutathione to produce glutamate, a mechanism indispensable for maintaining MDSC suppressive potency. Experimental evidence suggests that this pathway is related to EL4 lymphoma progression (125). Recent functional assays further reveal that inhibiting glutamine metabolism in myeloid cells can induce the conversion of MDSCs into inflammatory macrophages with antigen-presenting capabilities, thereby significantly enhancing the immunogenicity of the TME (126).

Metabolic reprogramming in CAFs is also closely linked to glutamine metabolism, which positions CAFs as a significant source of energy and metabolites for cancer cells and the TME. Their elevated glycolysis and glutaminolysis provide α-KG, acetate, and various amino acids to fuel the microenvironment (127). Studies on B-lymphoma revealed that CAFs exhibit higher GLS activity, and inhibiting GLS can induce CAF transformation and functional downregulation (128). Interestingly, glutamine deficiency in the TME induces macrophage-like features in CAFs (129). Glutamine deprivation triggers macropinocytosis in CAFs, enabling the uptake and degradation of protein into amino acids. This provides carbon and nitrogen sources for both CAFs and pancreatic ductal adenocarcinoma (PDAC) cells, maintaining stromal cell function and tumor cell metabolism to promote tumor growth and fibrosis. However, glutamine regulation of CAFs in HMs has not been comprehensively investigated, representing a potential future direction.

The evolution of HMs relies deeply on the metabolic support provided by adipocytes, with glutamine serving as the central link in this inter-cellular communication.Similar to CAFs, adipocytes are crucial cells that supply glutamine to tumors for energy metabolism and enhance therapy resistance, particularly significant in HMs. Enhanced GS expression was observed in adipocytes in studies of ALL and AML. It not only provides carbon and nitrogen sources for TACs but also reinforces survival defense mechanisms across the microenvironment by regulating autophagy levels (130, 131). Adipocyte-derived glutamine can activate mTORC1 signaling in surrounding cells, enhancing their anti-apoptotic capacity under chemotherapeutic stress. This emergence of glutamine metabolism-associated resistance has also been identified in MM (132). This metabolic reprogramming, orchestrated by non-malignant cells, constructs a highly stable metabolic barrier that allows TACs to continuously support tumor evolution.

#### 2.3.2 Arginine

Arginine metabolic reprogramming is a core metabolic node driving immune evasion within the HM-TME. Within the TME, arginine degradation is primarily regulated by two key enzymes: ARG1 and iNOS. ARG1, predominantly expressed by tumor-associated myeloid cells, such as M2 macrophages and MDSCs, catalyzes the hydrolysis of arginine into ornithine and urea. Conversely, iNOS, mainly derived from M1 macrophages, metabolizes arginine into citrulline and NO. As an essential amino acid for T-cell activation and effector function, ARG1-mediated arginine depletion directly suppresses T cell activation and proliferation, establishing a metabolic barrier to anti-tumor immunity. For example, in AML, ARG1-overexpressing myeloid cells (predominantly PMN-MDSCs) suppress T-cell proliferation and cytotoxic activity by consuming arginine (133). This suppression has clear immunological consequences: arginine deficiency triggers the downregulation of the CD3 ζ-chain in the T-cell receptor (TCR) complex, impairing signal transduction and inducing G0-G1 cell cycle arrest (134). In MDS, MDSCs significantly suppress the cytotoxic activity of CD8+ T cells through the STAT3-ARG1 pathway, establishing a direct link between metabolic nodes and immune exhaustion (135). Interestingly, ARG1 expression in MDSCs and TAMs is not static but dynamically regulated by cytokines and tumor cell signals within the TME. For instance, in AML, tumor cell-secreted MUC1 has been identified as a key factor driving MDSC expansion and ARG1 overexpression, thereby exacerbating T-cell suppression (136). Similarly, in DLBCL, high expression of TREM2, a classic anti-inflammatory M2 polarization marker, on circulating myeloid MDSCs correlates with poor prognosis. TREM2 promotes ARG1 expression in M-MDSCs; its arginine metabolism depletes arginine, ultimately suppressing T cell proliferation (137). These findings highlight that arginine metabolic reprogramming is a prevalent phenomenon across diverse TME cell types. Furthermore, the ARG1-mediated immunosuppression is a highly targetable process driven by signaling pathways unique to HMs.

Arginine metabolism also shapes the TME through the accumulation of metabolites. Ornithine decarboxylase 1 (ODC1)is the key enzyme catalyzing ornithine to polyamines (e.g., putrescine, spermidine). In DLBCL, ODC1 overexpression in macrophages inhibits M1 polarization and promotes M2 polarization, ultimately fostering an “immune-deserted” microenvironment (138). Specifically, ODC1 overexpression in DLBCL cells activates pathways like Wnt/β-catenin and PI3K-AKT, promoting secretion of cytokines (IL-4, IL-10, IL-13, TGF-β), thereby driving macrophage M2 polarization and facilitating immune evasion via an “immune-deserted” microenvironment (138). Recent evidence indicates that TAMs utilize tumor-derived arginine for polyamine synthesis, which promotes pro-tumor polarization through TDG-mediated epigenetic modifications, thereby facilitating immune evasion (139). Dual arginase inhibitors (e.g., OATD-02) have shown experimental potential in reprogramming the TME by restoring arginine availability and reducing polyamine accumulation, thus enhancing anti-tumor immunity (140).

Concurrently, NO generated by iNOS pathway, which competes with ARG1 for arginine, regulates TME metabolism with highly concentration-dependent effect. In HMs, myeloid-derived NO can induce the formation of functional Tregs and impair NK cell function, specifically inhibiting Fc receptor-mediated antibody-dependent cellular cytotoxicity (ADCC) (141, 142). Research on NO-mediated TME alterations in HMs remains poorly understood, representing an area that requires further in-depth investigation.

Beyond its core metabolic pathways, arginine is central to important epigenetic modifications. Protein arginine methyltransferases (PRMTs) catalyze methylation modifications on arginine residues of substrate proteins. Through substrate modification, they broadly regulate gene transcription, RNA splicing, and metabolic pathways, playing key roles in tumorigenesis and progression. For instance, in mouse leukemia models, PRMT1 induces M2 macrophage polarization and angiogenesis via the FGF2/PI3K/Akt pathway (143). Another enzyme, peptidylarginine deiminase 4 (PAD4) catalyzes citrullination of arginine residues on histones and non-histone proteins play a crucial role in NET formation. Blocking this process inhibits NETosis, thereby delaying disease progression and alleviating immune suppression within the microenvironment in MM and acute promyelocytic leukemia (APL) (144, 145). PRMTs, particularly PRMT5, have emerged as promising therapeutic targets, with several inhibitors currently in clinical trials for HMs.

In conclusion, arginine metabolic reprogramming in TACs of HMs is not merely an isolated biochemical event but a driver of T-cell exhaustion and macrophage polarization through core nodes such as ARG1-mediated depletion, polyamine synthesis, and NO production. This rewiring creates a nutrient-depleted, immunosuppressive ecosystem that facilitates tumor immune escape. Targeting these key metabolic vulnerabilities—through strategies like arginase inhibitors, polyamine metabolism modulators, or PRMT inhibitors—represents a promising and rational strategy to enhance the efficacy of immunotherapy and overcome treatment resistance in hematologic cancers. Targeting these key metabolic nodes represents a promising strategy to enhance the efficacy of immunotherapy in HMs.

#### 2.3.3 Tryptophan

In the TME of HMs, tryptophan metabolism reprogramming is a central mechanism regulating immune evasion. Tryptophan is degraded via at least three known pathways: the IDO1-oxidative pathway, the IL4I1-oxidative pathway, and the serotonin pathway (146). Kynurenine (Kyn) is a pivotal metabolite derived from tryptophan catabolism. Within the TME, the kynurenine production pathway initiated by IDO1 primarily influences TME immunosuppression, which is the focus here.

High IDO1 expression in tumor-associated myeloid cells is a prominent feature in various HMs and correlates with poor prognosis. For instance, in CLL, tumor cells induce high IDO1 expression in CD14+ HLA-DRlo MDSCs. And these cells not only inhibit T cell proliferation and but also promote the generation of Treg, thereby constructing a potent immunosuppressive network (147). This finding reveals that IDO1 is not merely a metabolic enzyme but a critical tool employed by TACs to cripple the anti-tumor immune response.

The immunosuppressive function of IDO1 is primarily mediated by its metabolite, kynurenine, which is the primary metabolite of the IDO1-oxidative tryptophan pathway. As a key signaling molecule, kynurenine profoundly alters the function of immune cells within the TME by binding to its receptor, the AhR. In the TME of AML, MSCs, stimulated by tumor-cell-secreted IFN-γ, upregulate IDO1. The resulting kynurenine production promotes Treg infiltration via the AhR signaling pathway, thus attenuating the host’s anti-tumor capacity (148). More compelling evidence shows that the kynurenine–AhR axis directly acts on myeloid cells, driving their differentiation towards an immunosuppressive phenotype. In various tumor models, kynurenine has been shown to activate AhR in macrophages, promoting their polarization towards an M2-like, pro-tumoral phenotype characterized by upregulation of CD206 and PD-L1, downregulation of MHC class I/II and co-stimulatory molecules, and acquisition of a potent CD8+ T cell-suppressive function (149). Concurrently, AhR activation has been demonstrated to directly induce the massive mobilization and expansion of MDSCs and enhance their immunosuppressive activity, a process involving the regulation of chemokine receptors like CXCR2 (150). These studies clearly link tryptophan metabolism, AhR signaling, and myeloid-cell-mediated immunosuppression, highlighting its crucial role as a core metabolic checkpoint.

Furthermore, the kynurenine-AhR axis can directly impair the function of cytotoxic immune cells. In the TME of AML, this inhibition is mediated by kynurenine binding to its primary receptor, the AhR, which triggers downstream pathways such as AhR-NF-κB (151). Research indicates that kynurenine can inhibit the expression of activating receptors (e.g., NKp46 and NKG2D) on NK cells, thereby weakening their direct killing ability against tumor cells. This is particularly critical in malignancies like AML, which rely on NK cell surveillance (152, 153).

Beyond the major amino acids, the reprogramming of others like L-phenylalanine and histamine also modulates tumor-associated cell function. In T-ALL, L-phenylalanine can inhibit PKM2 activity in MDSCs, reducing their glycolytic flux and immunosuppressive ROS production, thereby promoting their differentiation and reducing tumor burden (154). Similarly, in AML, histamine (derived from histidine) signaling via H2 receptors can inhibit NADPH oxidase 2 (NOX2) activity in myeloid cells, diminishing ROS-mediated suppression and alleviating intratumoral immunosuppression (155).

**Figure 3:**
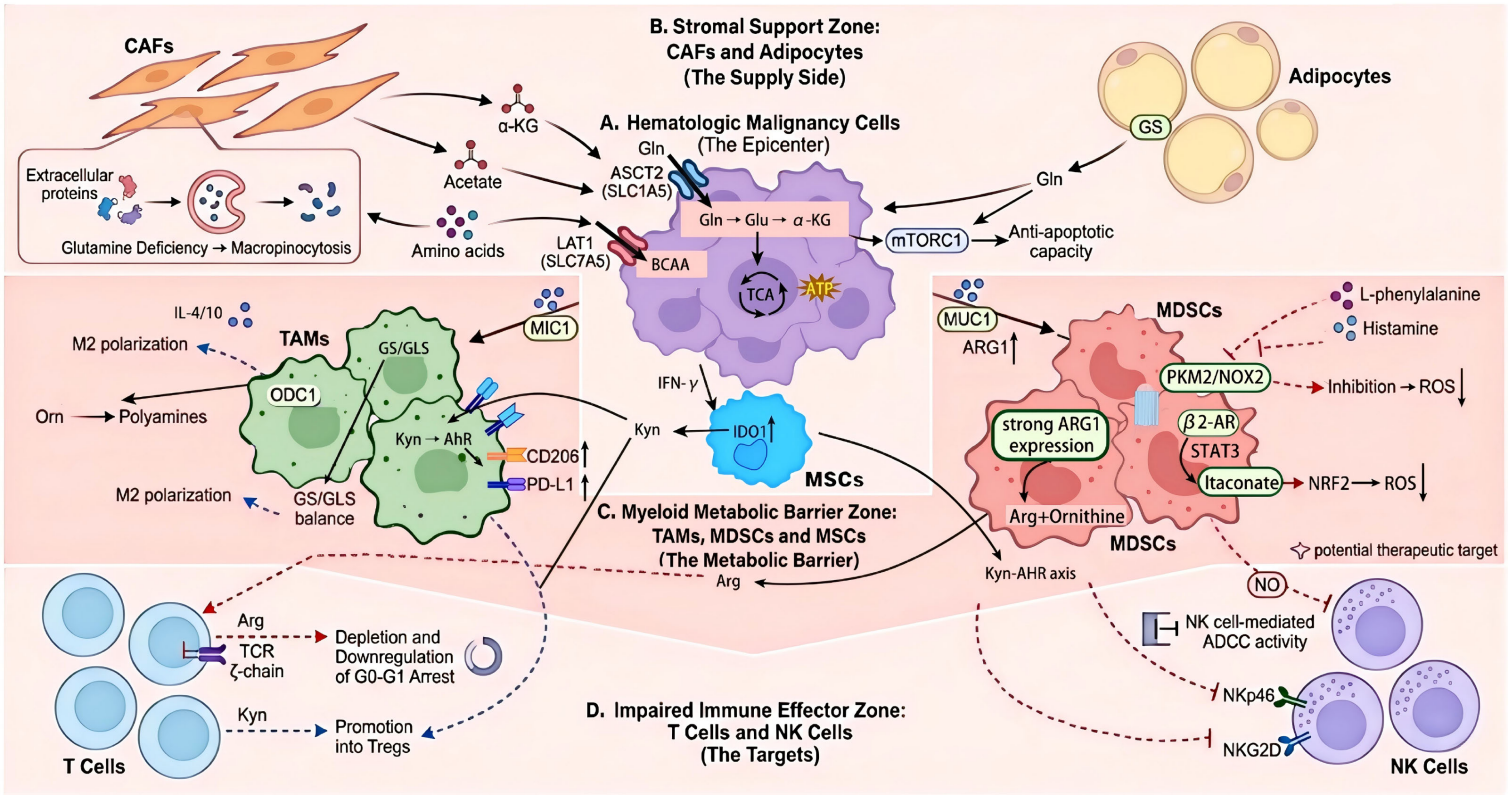
The metabolic ecological network of amino acids in the hematologic malignancy tumor microenvironment (HM-TME). This schematic illustrates the complex, multi-compartmental metabolic crosstalk centered on key amino acids—glutamine, arginine, tryptophan, and branched-chain amino acids (BCAAs)—within the HM-TME. It depicts how symbiotic and competitive interactions between malignant cells, stromal supporters, and immunosuppressive myeloid cells collectively orchestrate a metabolic landscape that cripples anti-tumor effector immunity. **(A)** Malignant cells as the metabolic epicenter. Leukemia, lymphoma, or myeloma cells drive the ecosystem by avidly importing exogenous glutamine (Gln) via alanine, serine, cysteine-rich transporter 2 (ASCT2/SLC1A5) and BCAAs via L-type amino acid transporter 1 (LAT1/SLC7A5). Intracellularly, glutaminase (GLS) converts Gln to glutamate (Glu), which is further metabolized to alpha-ketoglutarate (α-KG) to fuel the tricarboxylic acid (TCA) cycle for energy (adenosine triphosphate, ATP) production and biosynthesis. These cells also secrete cytokines (e.g., IL-4, IL-10) that instruct the surrounding microenvironment. **(B)** Stromal cells as metabolic supporters. Cancer-associated fibroblasts (CAFs) utilize macropinocytosis to scavenge nutrients under glutamine deprivation. Adipocytes upregulate glutamine synthetase (GS) to synthesize and secrete Gln, which activates the mammalian target of rapamycin complex 1 (mTORC1) pathway in adjacent malignant cells, enhancing their survival. Mesenchymal stem cells (MSCs), as key components of the stromal support zone, are regulated by tumor-derived IFN-γ to upregulate IDO1 expression, thereby supplying large amounts of the immunosuppressive metabolite Kyn to the myeloid metabolic barrier zone and impaired immune effector zone. **(C)** Myeloid cells establish a metabolic barrier. Tumor-associated macrophages (TAMs) and myeloid-derived suppressor cells (MDSCs) catabolize amino acids to enforce immunosuppression. TAMs integrate multiple pathways: arginine metabolism via ornithine decarboxylase 1 (ODC1) supports M2 polarization; glutamine metabolism balances GS and GLS activity; and tryptophan catabolism to kynurenine (Kyn) activates the aryl hydrocarbon receptor (AhR), upregulating M2 markers (CD206) and programmed death-ligand 1 (PD-L1). MDSCs deplete arginine via high arginase-1 (ARG1) expression and adapt glutamine metabolism to survive oxidative stress. Notably, L-phenylalanine and histamine can suppress the pyruvate kinase M2 (PKM2)/NADPH oxidase 2 (NOX2) axis to reduce reactive oxygen species (ROS), preserving MDSC function. **(D)** Immune effector dysfunction. The resulting metabolic landscape directly impairs anti-tumor lymphocytes. In T cells, arginine depletion causes T cell receptor (TCR) ζ-chain downregulation and cell cycle arrest, while Kyn promotes regulatory T cell (Treg) expansion. Natural killer (NK) cells show reduced expression of activating receptors (e.g., NKp46, NKG2D) and diminished cytotoxicity, including antibody-dependent cell-mediated cytotoxicity (ADCC), further suppressed by myeloid-derived nitric oxide (NO). Abbreviations and symbols: Purple irregular clusters, malignant cells (e.g., acute myeloid leukemia/AML, diffuse large B-cell lymphoma/DLBCL, multiple myeloma/MM); Orange spindle shapes, CAFs; Large yellow circles, adipocytes; Blue amorphous cells, MSCs; Green amorphous shapes, TAMs; Red amorphous shapes, MDSCs; Blue circles, T cells; Purple circles, NK cells; Arrows, amino acid transport/metabolic flow. All abbreviations are defined in the legend text.

In summary, amino acid metabolic reprogramming in the HM-TME enforces a tolerogenic state through nutrient deprivation and disruption of effector immunity. Glutamine bidirectionally regulates macrophage polarization, while its role in polarizing TAMs in HMs requires further definition. Beyond macrophages, glutamine metabolism is critically linked to the survival and functional persistence of MDSCs and serves as a key metabolic substrate provided by CAFs and adipocytes to fuel tumor growth. Arginine depletion inhibits T cell function, and its metabolites influence macrophage polarization. Tryptophan catabolism induces Treg expansion and M2-TAM polarization via the IDO1/TDO2-Kyn-AhR axis, creating profound immunosuppression. Metabolites like phenylalanine and histamine can impair MDSC function. Collectively, this rewired metabolism systemically weakens anti-tumor immunity by depleting essential amino acids and generating inhibitory metabolites, presenting a network of therapeutic vulnerabilities (**Figure 3**).

## 3 Metabolic crosstalk network: novel strategies for targeted intervention

Crosstalk is a prevalent phenomenon in the TME of HMs, representing a crucial process whereby cellular and non-cellular components mutually influence each other (156). Metabolic rewiring is a key component of this interplay, serving as both a driver and a consequence of cellular communication. The utilization of metabolites involves extensive crosstalk between tumor cells and TACs, encompassing metabolic synergy, nutrient competition, and bidirectional communication.

Metabolites such as lactate, fatty acids, glutamine, and arginine constitute core nodes within this metabolic network. Lactate, sustained by the mutual reinforcement of the Warburg effect and the reverse Warburg effect, fuels the TME. Polarized macrophages can further exacerbate tumor glycolysis, promoting tumor proliferation (157). Tumor cells induce aberrant fatty acid metabolism in BMAds via signaling molecules. This subsequently alters BMAds’ fatty acid release and adipokine secretion profiles, facilitating tumor invasion and immune evasion, thereby establishing bidirectional crosstalk. Glutamine, a vital energy substrate, is subject to competition for uptake between tumor cells and macrophages, and its metabolism is linked to tumor progression, providing a novel perspective for therapeutic intervention (158). Arginine depletion, mediated by ARG produced by immunosuppressive TAMs and MDSCs, disrupts T cell function. This multi-cellular synergy collectively constructs a pro-tumor metabolic network. Beyond these core metabolites, other substances such as kynurenine also play significant roles in TME metabolic rewiring and immune suppression.

Interventions targeting the metabolism of key substances have shown promise in solid tumors for synergistically delaying tumor progression and reversing treatment resistance (159). Therefore, targeting metabolic crosstalk holds significant potential for overcoming TME barriers in HMs, thus presenting a compelling rationale for further investigation toward clinical translation. Some of the targeting strategies mentioned below are summarized in **Table 1**.

**Table 1:** Overview of key pathways and potential drugs for metabolic targeted therapy in the hematologic malignancy microenvironment.

Metabolic type	Associated cells	Disease type	Potential target/pathway	Potential or approved therapeutics	Clinical status	References
Glucose Metabolism	TAMs	DLBCL	BCL-3/NF-κB	NF-κB inhibitor (IKK16)	Preclinical	(25)
Glucose Metabolism	Tumor cells	MM	USP5-STAT2- PFKFB4	USP5 inhibitor	Preclinical	(160)
Glucose Metabolism	TAMs	HL	PI3K/AKT	RP6530 (Tenalisib)	Phase II	(41, 161)
Glucose Metabolism	FRCs	DLBCL	IL-2 signaling pathway	FAP-IL2v (Simlukafusp alfa)	Phase II	(162, 163)
Glucose Metabolism	FRCs	DLBCL	4-1BB signaling pathway	FAP-4-1BBL (RO7122290)	Phase I	(162, 164)
Glucose Metabolism	TAMs	DLBCL	PDPK1-PGK1 pathway	OSU-03012	Preclinical	(157)
Glucose Metabolism	Tumor cells	DLBCL	GSK3β/β- catenin/c-Myc	ENO2 inhibitor	Preclinical	(39)
Glucose Metabolism	Tumor cells	NHL/MM	MCT1	AZD3965	Phase I/II	(46, 57, 165)
Glucose Metabolism	Tumor cells	CLL	TLR9/IκBζ	Dimethyl itaconate (DI)	Preclinical	(69)
Glucose Metabolism	Tumor cells	Lymphoma	IRG1-itaconate- TFEB axis	Thimerosal	Preclinical	(68)
Glucose Metabolism	MDSCs	Hematologic malignancies	JAK/STAT3	JSI-124	Preclinical	(65)
Glucose Metabolism	MDSCs	Hematologic malignancies	Mitochondrial ETC	Rosiglitazone, Rotenone	Preclinical	(65)
Glucose Metabolism	Tumor cells	Hematologic malignancies	ACOD1 (IRG1)	Citric acid	Preclinical	(166)
Lipid Metabolism	TAMs	MM	PPARγ-FABP5-Carnitine palmitoyltransferase 1A (CPT1A)	FABP5 inhibitor (BMS309403), CPT1A inhibitor (Etomoxir)	Preclinical	(79)
Lipid Metabolism	TAMs/B cells	B-cell lymphoma	sPLA2-driven lipid metabolism	sPLA2 inhibitor (Varespladib)	Preclinical	(101)
Lipid Metabolism	MDSCs	B-cell precursor ALL	FAO	Etomoxir, Ranolazine	Preclinical	(167)
Amino Acid Metabolism	TAMs	AML/MDS	Glutaminolysis	CB-839 (Telaglenastat)	Phase I/II	(168)
Amino Acid Metabolism	Tumor cells	AML	IDH/IDH mutation	Ivosidenib/Enasidenib	Approved	(169, 170)
Nucleotide Metabolism	TAMs/Tumor cells	CLL/MM	CD38-NAD+ metabolism	Daratumumab	Approved	(171)
Tryptophan Metabolism	MDSCs	AML	IDO1 pathway	Epacadostat	Phase II	(172)
Other	Tumor cells/TME	AML	MerTK/FLT axis	MRX-2843	Phase I	(173)

### 3.1 Targeting glycolytic metabolic reprogramming

Glycolytic metabolic reprogramming extensively affects various cells within the TME, and its metabolites, including lactate and itaconate, play key roles in tumor progression. Utilizing drugs targeting lactate metabolism can significantly improve the TME, primarily categorized as follows:

1. Targeting Lactate Production: While broadly targeting glycolysis (e.g., via PI3K/AKT pathways) holds promise, its systemic application is limited by on-target toxicity to normal hematopoietic and immune cells. Recent evidence indicates that AML cells can achieve “metabolic escape” by upregulating lactate utilization pathways when challenged with BET inhibitors, thereby conferring drug resistance (174). Consequently, targeting lactate production serves a dual purpose: inhibiting tumor bioenergetics and disrupting these adaptive resistance mechanisms. In MM cells, two pathways mediated by ubiquitin-specific protease 5 (USP5), namely the USP5/c-Maf pathway and the USP5-STAT2-PFKFB4 pathway, have been demonstrated as therapeutic targets; the USP5 inhibitor mebendazole shows preclinical efficacy (160, 175). Similarly, in DLBCL, Utilizing the FAP-targeted 4-1BB agonist (FAP-4-1BBL, RG7827) and the IL-2 variant immunocytokine (FAP-IL2v, simlukafusp alfa) to target the 4-1BB signaling pathway and the IL-2 signaling pathway, respectively, both directly remodel the immunosuppressive CAF niche, demonstrating a stromal-targeted approach unique to lymphoid malignancies (162). The key limitation and future direction lie in minimizing BM suppression while effectively starving tumors of lactate, requiring more selective drug delivery or combination regimens that protect hematopoietic stem cells.

2. Targeting Lactate Transport: Lactate transporters MCT1/MCT2 are central to the metabolic crosstalk between hematologic tumor cells and immune effectors. In MM, pharmacological inhibition of MCT1/4-mediated lactate trafficking significantly restores sensitivity to proteasome inhibitors (e.g., bortezomib) and orchestrates a more favorable immune microenvironment within the BM (174). Inhibiting major lactate transporters MCT1/MCT2 can reverse lactate-mediated immunosuppression, notably in MDSCs which are abundant in hematologic TMEs (176). This strategy is particularly relevant for myeloid malignancies and lymphomas where MDSCs contribute to therapy resistance. Furthermore, studies confirm that Notch-mediated activation of MCT1/2 is critical for maintaining their immunosuppressive phenotype; targeting this axis effectively reverses T-cell metabolic suppression (46, 57, 176).

3. Targeting Downstream Metabolite Signaling: Targeting histone lactylation represents a promising strategy. Miao Zhu et al. revealed HNRNPH1’s role in lactylation-dependent M2 macrophage polarization (177). Thus, epigenetic targeting warrants increased attention. Targeting itaconate to remodel the immune microenvironment is equally valuable. Beyond conventional JAK/STAT3 inhibition, suppressing the electron transport chain (ETC) to block itaconate synthesis emerges as an attractive approach. Critically, itaconate-activated NRF2 in MDSCs mitochondria correlates with chemotherapy resistance in HMs (65).

### 3.2 Targeting lipid metabolic reprogramming

Lipid metabolic reprogramming occurs extensively across cell types. In HMs, lipid metabolic reprogramming serves as a pivotal mechanism for tumor cells to adapt to specialized niches, such as the BM, and acts as a central driver of immune evasion and drug resistance. Unlike solid tumors, HMs exhibit unique metabolic crosstalk with BMAds. Targeting strategies primarily focus on fatty acids and LPA. The ultimate goal of these strategies is to disrupt the energy supply and signaling pathways that tumor cells rely on for proliferation and survival.

Targeting fatty acids: Emerging evidence suggests that BMAds facilitate leukemia progression by secreting free fatty acids (FFAs) and upregulating the fatty acid translocase CD36 on the surface of malignant cells, thereby providing essential energetic substrates that sustain survival under chemotherapeutic stress (178, 179). Especially, CD36 is overexpressed in AML stem cells and imatinib-resistant chronic myeloid leukemia (CML) clones. Experimental studies have demonstrated that blocking CD36 with monoclonal antibodies or small-molecule inhibitors (e.g., SMS121) effectively reduces the lipid burden of leukemic cells and reverses resistance to tyrosine kinase inhibitors (TKIs) or hypomethylating agents (HMAs) (180, 181). Furthermore, studies confirm that palmitic acid reprograms TAMs by activating the ALOX12 pathway and inhibiting AMPK signaling, thereby reversing their mediation of chemoresistance in MM. This suggests a novel strategy to sensitize cancer cells to chemotherapy by targeting the palmitic acid-ALOX12-AMPK axis (83).

Targeting LPA signaling pathway: The functional heterogeneity of the LPA signaling pathway within the HM-TME also warrants careful consideration. LPA binds to receptors such as LPA2 to participate in inflammatory responses and cancer metabolic reprogramming. In cancer, LPA has been demonstrated to play roles in mediating the secretion of amphiregulin (AREG) by CAFs to enhance tumor invasiveness (182). In CLL and B-cell lymphomas, LPA primarily acts through its receptors (e.g., LPA2) to trigger the AKT/PKB signaling axis and induce the expression of VEGF, thereby promoting anti-apoptotic effects and angiogenesis (142, 174). Building upon the mechanistic understanding of the LPA-AKT-VEGF axis, recent preclinical studies have underscored the therapeutic potential of targeting the ATX-LPA signaling cascade to disrupt the supportive hematologic TME. For instance, in T-cell lymphoma models, pharmacological antagonism of LPA receptors using Ki16425 has been shown to significantly impede tumor progression by concurrently inducing apoptosis and suppressing glycolytic metabolism through the downregulation of glucose transporter 3 (GLUT3) and MCT1 (183). Beyond direct tumor cell inhibition, the ATX-LPA axis serves as a critical “lipid-regulated immune checkpoint” within the microenvironment. Elevated LPA levels engage the LPAR5 receptor on CD8+ T cells, leading to the metabolic exhaustion and functional suppression of these effector cells, thereby facilitating immune evasion (184). Intriguingly, Dapeng Zhang et al. report that LPA in colorectal cancer induces M1 macrophage polarization and promotes T-cell infiltration, thereby restraining tumor progression (185), while it predominantly functions as a pro-survival factor within the BM niche of HMs. This divergence underscores the necessity of tailoring therapeutic interventions to the specific anatomical and cellular context of the disease, and warrants further investigation.

### 3.3 Targeting amino acid metabolic reprogramming

Amino acid metabolic reprogramming, akin to glucose and lipid pathways, primarily reshapes the TME and fosters an immunosuppressive microenvironment. Among targeted pathways, glutamine and arginine metabolism currently receive significant research attention.

Disrupting glutamine metabolism impairs metabolic-immune crosstalk: In the AML TME, CD34^+^ pre-B cells critically rely on glutamine to activate the MIF-(CD74+CD44) signaling axis, accelerating tumorigenesis.These findings underscore the therapeutic potential of dual-targeting glutamine metabolism and the MIF pathway, providing a mechanistic rationale for combined pharmacological interventions to disrupt the pro-tumorigenic niche (186). Furthermore, the crosstalk between asparagine and glutamine in MSCs is closely associated with chemoresistance. In acute ALL, leukemic blasts exhibit a profound auxotrophy for asparagine. Experimental evidence has demonstrated that ALL blasts actively “educate” neighboring BM mesenchymal MSCs by secreting glutamine. Upon receiving glutamine signals, MSCs upregulate asparagine (Asn) synthesis via GS and asparagine synthetase (ASNS), subsequently extruding it into the extracellular space through the SNAT5 transporter (187). This blast-driven metabolic trade-off significantly counteracts the efficacy of L-asparaginase (L-ASNase). Consequently, developing inhibitors targeting GS or SNAT5 in MSCs to disrupt this stromal-to-blast nutritional support represents a specific strategy to overcome L-ASNase resistance (187, 188).

Targeting arginine metabolism: Depletion of arginine is a hallmark of the immunosuppressive landscape in HMs. In the TME of AML and MM, G-MDSCs consume extracellular arginine by releasing high levels of Arg1. Studies have proven that an arginine-depleted environment induces cell cycle arrest and impairs the effector functions of T and NK cells (133, 189, 190). Cumulative evidence indicates that combining L-arginine with chemotherapy enhances therapeutic efficacy (191), positioning its repletion as a promising adjunctive strategy. Experimental application of NOHA (an ARG1 inhibitor) and L-NMMA (an iNOS inhibitor) significantly reduced AML blasts’ immunosuppressive effects on T cells (133). Additional pharmacological ARG-targeting agents include melatonin (192) and CB1158 (193). The novel oral arginase inhibitor CB-1158 has shown the capacity to reverse myeloid-mediated T-cell suppression in ex vivo experiments. In MM models, it has also been found to mitigate chemotherapy-induced adverse effects (e.g., from bortezomib) while synergistically enhancing anti-tumor activity (194, 195). This strategy’s advantage lies in its ability to simultaneously target suppressive cells and protect effector immune function, offering high translational potential.

Targeting tryptophan metabolism: Tryptophan catabolism via the IDO/TDO pathway produces kynurenine, a key inducer of immune tolerance. In DLBCL and CLL, IDO1 is significantly upregulated in TAMs and Tregs (196, 197). However, preclinical studies (e.g., in the Eµ-TCL1 CLL model) have revealed that targeting IDO1 alone (using agents like Epacadostat) often yields limited efficacy due to the compensatory expression of IDO2 or TDO2 within the TME (198). This finding underscores the necessity of multi-target metabolic blockade (e.g., dual IDO1/TDO2 inhibition) to address complex metabolic redundancies in HMs.

## 4 Challenges and perspectives

Metabolic reprogramming within TACs of the HM-TME has emerged as a central paradigm for understanding tumor progression and immune evasion. The reconstructed metabolic homeostasis of glucose, lipid, and amino acid pathways forms a complex interactive network. Key metabolites, such as lactate and fatty acids, actively reshape the immunosuppressive landscape by driving the polarization of TACs and modulating overall TME composition, thereby presenting critical therapeutic targets. However, current research in this field remains nascent and faces several major challenges.

Firstly, metabolic heterogeneity in TACs is inadequately characterized. Existing investigations have predominantly focused on mainstream immune populations like TAMs and MDSCs, often neglecting the distinct metabolic features of other cellular components within the niches (166, 167). Comprehensive explanations linking specific metabolic profiles to prognostic outcomes across this diverse cellular ecosystem are still lacking. Furthermore, metabolic variations among different HMs and their shared microenvironmental alterations require deeper exploration, an area where research currently lags behind that of solid tumors. Beyond core pathways, the immunoregulatory mechanisms of novel metabolic modifications, such as protein lactylation, remain unclear. Another critical issue is the existence of contradictory findings regarding the role of certain metabolites (e.g., reactive oxygen species, ROS), suggesting that context-dependent answers may need to be sought across different cancer types. Additionally, while viruses are known to promote certain HMs, their specific role in remodeling TAC metabolism to sustain tumor growth is poorly understood (199). Future studies must leverage advanced technologies like single-cell metabolomics to decipher these malignancy- and cell-type-specific metabolic mechanisms.

Secondly, the inherent “double-edged sword” effect of clinical interventions remains unavoidable. Targeting metabolic reprogramming within the TME represents a transformative therapeutic frontier for HMs. However, the unique anatomical localization of these cancers—primarily within the BM—presents a formidable challenge: avoiding myelotoxicity. The BM serves as both a sanctuary for malignant cells and the essential niche for normal HSPCs. Given the significant metabolic overlap between malignant clones and healthy hematopoietic cells, interventions targeting glucose, lipid, or amino acid metabolism often incur “collateral damage, “ leading to severe myelosuppression. For instance, glycolytic inhibitors (e.g., targeting LDHA or using 2-DG) can suppress T-cell acute lymphoblastic leukemia (T-ALL) or AML but concurrently induce anemia and neutropenia, as stressed HSPCs themselves rely on glycolysis (200–202). Similarly, L-asparaginase exerts BM toxicity partly through its GLS side activity; glutamine depletion disrupts nucleotide synthesis in hematopoietic precursors and immune cells, causing metabolic exhaustion and cytotoxicity (203, 204).

To navigate these challenges, several promising strategies are being developed. Dosage optimization and combination with supportive therapies show clinical potential. The development of next-generation asparaginases with minimal GLS activity exemplifies this approach, maintaining anti-tumor efficacy while significantly reducing myelotoxicity (203). Furthermore, identifying “metabolic windows” through real-time metabolomic monitoring could allow for timed drug administration when tumor metabolic demand peaks, supplemented with hematopoietic growth factors or metabolic intermediates (e.g., pyruvate) to protect normal hematopoiesis (205, 206).

Advanced delivery systems are crucial for enhancing specificity. Targeted delivery using bone-homing ligands (e.g., alendronate) or BM-specific nanocarriers can concentrate metabolic inhibitors within tumor-rich niches, minimizing exposure to healthy hematopoietic regions (207). Nanomedicine, in particular, paves new avenues for TME-targeted therapy (208–210). For example, in T-ALL, a designed Metabolic Reprogramming Immunosurveillance Activating Nanomedicine (MRIAN) can degrade to release L-phenylalanine, targeting MDSC-associated ROS to restore immunosurveillance (209, 210). Similarly, spermidine-metal-immunopeptide nanocomplexes have been shown to promote damage-associated molecular pattern (DAMP) release in lymphoma models, enhancing immune recognition and immunosurveillance (211). The development of sophisticated carriers and targeting moieties enables precise targeting of TACs, facilitating strategies like TAM re-education and hypoxia amelioration (212–217). However, the advancement of such nanomedicines remains fundamentally dependent on a deeper mechanistic understanding of the TME.

Finally, integrating metabolic modulation with immunotherapy, especially chimeric antigen receptor (CAR)-T therapy, holds great promise (218). The efficacy of CAR-T cells in HMs is often limited by TME-imposed metabolic barriers. Strategies to metabolically engineer the TME or the CAR-T cells themselves are being explored. For instance, modulating arginine levels has been proposed to improve CAR-T-mediated leukemic clearance (219). In other cancer models, engineered CAR-T cells have shown an ability to suppress MDSC recruitment via CXCR4 targeting, suggesting a pathway to evade MDSC suppression (161, 220). Future development of targets mitigating CAR-T exhaustion in TACs will be crucial. A notable example of multidisciplinary integration is the combination of CAR-T with chemo-photothermal nanotherapy, which has demonstrated enhanced efficacy in non-Hodgkin lymphoma (NHL) models (163). This exemplifies the pivotal future direction of combining modalities.

While significant challenges remain—particularly regarding metabolic heterogeneity and therapeutic specificity—the path forward lies in precision strategies guided by detailed metabolic profiling. Leveraging advanced delivery technologies, intelligent combination therapies, and multidisciplinary integration will be key to breaking current bottlenecks and enabling effective, patient-tailored interventions in HMs.

## 5 Conclusions

This systematic review elucidates that metabolic reprogramming constitutes a fundamental and pervasive hallmark of the HM-TME. Crucially, this rewiring extends beyond the autonomous adaptation of malignant cells to encompass a dynamic, interconnected network of stromal and immune cells—including TAMs, MDSCs, CAFs, and BMAds. Through coordinated alterations in glucose, lipid, and amino acid metabolism, these TACs collectively engineer an immunosuppressive and pro-tumorigenic niche that fuels disease progression and confers therapy resistance. Targeting these key metabolic nodes shows significant therapeutic potential. However, major challenges persist, including insufficient characterization of metabolic heterogeneity within the TME and clinical limitations such as treatment-related toxicity. Future integration of single-cell metabolomics with advanced strategies like nanocarrier-based delivery and metabolic-immunotherapy combinations is crucial to reverse the pro-tumorigenic properties of the microenvironment and advance clinical translation.
